# Impact of Duffy polymorphisms on parasite density in Brazilian Amazonian patients infected by *Plasmodium vivax*

**DOI:** 10.1186/s12936-019-2918-4

**Published:** 2019-08-27

**Authors:** Rechfy K. Abou-Ali, Anamika Dhyani, Alexandre L. Terço, Diana M. Toro, Katiane S. Gomes, Lucianna C. Tezza, Monique A. Negreiros, Celiane S. Batista, Márcia K. S. Souza, Edalton C. B. Sanguino, Sérgio R. L. Albuquerque

**Affiliations:** 1 University of the State of Amazonas, Manaus, Brazil; 2 Blood Center of the Amazon State, HEMOAM, Manaus, Brazil

**Keywords:** Alleles Duffy, *Plasmodium vivax*, Parasite density

## Abstract

**Background:**

The Duffy glycoprotein acts as the entry point for merozoites of *Plasmodium vivax* in the invasion of red blood cells. The host–parasite relationship has revealed new perspectives regarding the association between Duffy polymorphisms that can impact both the parasite density of this Plasmodium and the symptoms of this type of malaria. This study investigates the impact of Duffy polymorphisms on parasite density in patients infected with *P. vivax* in the Brazilian Amazon region.

**Methods:**

Genotypes and Duffy polymorphism allele frequencies were compared in 287 patients with malaria, presenting low, medium and high density of *P. vivax*. The diagnosis of malaria was performed using a specialized team with a standardized clinical-laboratory method, while the Duffy genotyping was performed through the Bead Chip BioArray system. Both teams are reference services in Brazil.

**Results:**

The FY*01 and FY*02 alleles were found in all three parasite density classes: low, medium and high, but when these alleles form genotypes with FY*02N.01 and FY*02W.01 alleles, they are found only in patients with low parasite density and low symptomatology. Another interesting finding found in this study is the presence of the genotype FY*02N.01/FY*02W.01 in one of the patients, presenting a very low parasite density and malaria considered subclinical, a genotype which had not been previously described in the literature.

**Conclusion:**

The presence of FY*02N.01 and FY*02W.01 alleles may have an impact on the reduction of clinical manifestations in malaria, leading to the development of subclinical malaria, making the infected individual an undetected natural reservoir, which may hinder the eradication of malaria in the Amazon.

## Background

Malaria is a major parasitic disease, affecting around 91 countries worldwide. In the year 2017, the World Health Organization (WHO) estimated 219 million cases of malaria worldwide, with about 435,000 deaths. In Brazil, 178,613 cases of malaria were registered, and the state with the highest number was the Amazonas with 77,344 cases. Among these, the infections caused by *Plasmodium vivax* predominated, with 82% of recorded cases. In recent years, a pattern of abnormal clinical complications associated with fatal infections caused by *P. vivax*, has been reported from Brazil [1, 2].

*Plasmodium vivax* is the species with the broadest geographic distribution and greatest prevalence in the world, except in the African continent, causing a debilitating illness affecting the quality of life and economic productivity of infected individuals. Malaria caused by *P. vivax* has been reported as benign and is rarely fatal, however, recent studies in Papua New Guinea, Indonesia, and Brazil, revealed, the existence of severe cases of malaria related to this Plasmodium. Infection can be followed by complications, such as, cerebral malaria, acute respiratory syndrome, liver dysfunction, severe thrombocytopenia and low weight in newborns derived from placental infection, symptoms that are more frequently observed in infections caused by *Plasmodium falciparum* [3, 4].

The Duffy blood group system is associated with invasion of reticulocytes by *P. vivax*. Studies in populations where malaria is common have demonstrated that erythrocytes that do not have Duffy antigens are relatively resistant to invasion by this Plasmodium. The antigens of the Duffy blood group system (Fy^a^, Fy^b^, Fy3, Fy5, Fy6) are encoded by two co-dominant allelic forms designated FYA (FY*01) and FYB (FY*02) from DARC (Duffy Antigen Receptor for Chemokines) gene which differ by a SNP (single Nucleotide Polymorphism) at position 125 of exon [5].

This difference is characterized by the exchange of guanine (G) by an adenine (A) (G125A), determining the expression of Fy^a^ and Fy^b^ antigens. The SNP at position-67 T>C in the GATA promoter region of the DARC gene, most frequently found, is characterized by the FY*02N.01 allele (*FYB*^**E**rythroid **S**ilent−ES^), Fy^b^ expression silencer in erythroid cells, determines the Fy (a− b−) phenotype, when in homozygous FY*02N, 01/FY*02N.01. Other less frequent nucleotide changes leading to the Duffy null phenotype have been described as shown in Table 1, although there are no studies correlating them with *P. vivax* susceptibility [6, 7].

**Table 1:** Names for FY (ISBT 008) blood group alleles

	Allele name	Nucleotide change
Phenotype		
FY:1 or Fy(a +)	FY*01 or FY*A	c. 125A>G
FY:2 or Fy(b +)	FY*02 or FY*B	
Null phenotypes		
Fy(a − b −) erythroid cells only	FY*01N.01	c.-67T>C
Fy(a − b −)	FY*01N.02	c.281_295del
Fy(a − b −)	FY*01N.03	c.408G>A
Fy(a − b −)	FY*01N.04	c.287G>A
Fy(a − b −)	FY*01N.05	c.327delC
Fy(a − b −)	FY*01N.06	c.395G>A
Fy(a − b −)	FY*01N.07	c.719delG
Fy(a − b −)	FY*01N.08	c.-69T>C
Fy(a − b −)	FY*01N.09	c.296_496delinsAGGCCACTG
Fy(a − b −) erythroid cells only*	FY*02N.01	c.-67T>C
Fy(a − b −)	FY*02N.02	c.407G>A
Fy(a − b −)	FY*02N.03	c.781G>A
Fy(a − b −)	FY*02N.04	c.179_180delCT
Fy(a − b −)	FY*02N.05	c.895G>A
Fy(a − b −)	FY*02N.06	c.151delT
Weak phenotypes		
Fy(a +^w^)	FY*01W.01	c.265C>T
Fy(a +^w^)	FY*01W.02	c.265C>T;c.298G>A
Fy(a +^w^)	FY*01W.03	c.680G>A
Fy(b +^w^), Fy^x^*	FY*02W.01	c.265C>T;c.298G>A
Fy(b +^w^), Fy^x^	FY*02W.02	c.145G>T;c.265C>T;c.298G>A
Fy(b +^w^)	FY*02W.03	c.266G>A
Fy(b +^w^)	FY*02W.04	c.901C>T

* Most frequent polymorphisms

http://www.isbtweb.org/fileadmin/user_upload/Working_parties/WP_on_Red_Cell_Immunogenetics_and/008_FY_Alleles_v4.1.pdf [10]

The FY*02W, 01 (*FYB*^**W**eak−W^) alelle, associated with weak Fy^b^ antigen expression are most often determined by c.256C>T and c.298G>A SNP, these polymorphisms occurring within the first intracellular loop of the Duffy protein with a frequency of approximately 2% in Caucasians. All polymorphisms of the duffy system found so far are described in Table 1, obtained from the website of the International Blood Transfusion Society—ISBT [8, 9].

Studies such as Nichols *et a.l* and Sellami et al. using flow cytometry technology have shown that individuals with two erythroid functional alleles (*FYA*/*FYA*) express approximately twice as much Fya antigens when compared to individuals with only one functional erythroid allele (*FYA*/*FYES* as well as low level of Fy^b^ antigen expression associated with *FYW* polymorphism [11, 12].

Tournamille et al. in an association study between the presence of *FYBES* alleles and Duffy glycoprotein expression, demonstrated that heterozygous carriers with a functional and a non-functional allele, as the following examples (*FYA*/*FYBES*, *FYB*/*FYBES*), express approximately only 50% of gpFy in their erythrocytes compared to homozygous individuals with both functional alleles (*FYA/FYA, FYB/FYB*), as shown in Table 2 [13].

**Table 2:** Expression of Duffy glycoprotein in the different Duffy phenotypes and genotypes

Alelos	Antígeno	Genótipo	Fenótipos	
			Fenotipagem	Expressão
*FYA*	Fy^a^	*FYA/FYA*	Fy (a + b −)	2 × Fya, 0 × Fyb
*FYB*	Fy^b^	*FYA/FYA* ^*ES*^	–	1 × Fya, 0 × Fyb
*FYX*	Fy^bweak^	*FYA/FYB* ^*ES*^	–	1 × Fya, 0 × Fyb
*FYA* ^*ES*^	–	*FYB/FYB*	Fy (a − b +)	0 × Fya, 2 × Fyb
*FYB* ^*ES*^	–	*FYB/FYB* ^*W*^	–	0 × Fya, 1,1 × Fyb
–	–	*FYB/FYA* ^*ES*^	–	0 × Fya, 1 × Fyb
–	–	*FYB/FYB* ^*ES*^	–	0 × Fya, 1 × Fyb
–	–	*FYB* ^*W*^ */FYB* ^*W*^	Fy (a − (b + fraco))	0 × Fya, 0,2 × Fyb
–	–	*FYB* ^*W*^ */FYA* ^*ES*^	–	0 × Fya, 0,1 × Fyb
–	–	*FYB* ^*W*^ */FYB* ^*ES*^	–	0 × Fya, 0,1 × Fyb
–	–	*FYA/FYB*	Fy (a + b +)	1 × Fya, 1 × Fyb
–	–	*FYA/FYB* ^*W*^	–	1 × Fya, 0,1 × Fyb
–	–	*FYA* ^*ES*^ */FYA* ^*ES*^	Fy (a − b −)	0 × Fya, 0 × Fyb
–	–	*FYA* ^*ES*^ */FYB* ^*ES*^	–	0 × Fya, 0 × Fyb
–	–	*FYB* ^*ES*^ */FYB* ^*ES*^	–	0 × Fya, 0 × Fyb

Adapted Zimmerman et al.

Yazdanbakhsh et al. demonstrated, by flow cytometry, a 10% reduction in Duffy protein in the red blood cell membrane of individuals with the *FYX* allele in heterozygosis, which is due to protein instability caused by the Arg89Cys mutation [14].

According to Zimmerman et al., the *FYBW* allele is associated with a reduced gpFy expression (approximately 10%) when compared to its expression from the *FYA* and/or *FYB* alleles [15].

Woolley et al. demonstrated in vitro studies differences in the level of expression of Duffy glycoprotein on reticulocyte surface of Caucasian and African–American individuals with Fy phenotype (a + b +) by flow cytometry. In their study, the expression level of the Fy^6^ epitopes, which is required for *Plasmodium vivax* invasion, was significantly lower in reticulocytes and mature red blood cells in the *FYB/FYB* genotypes than in the *FYA/FYA* or *FYA/FYB* genotypes. Therefore, the authors concluded that heterozygous individuals may have a greater amount of red cell receptor variations for *P. vivax*-binding parasitic proteins [16].

This study aims to investigate the impact of Duffy polymorphisms on parasite density in Brazilian Amazonian patients infected by *P. vivax.*

## Methods

In this study, individuals of any gender, age and skin color who were at the first infection reported by *Plasmodium vivax*, without co-infection with another Plasmodium species and without any associated disease, were randomly admitted.

Subjects were included according to the selection criteria of the research at the Tropical Medicine Foundation (TMF) Dr. Heitor Vieira Dourado, where blood samples were collected from January 2012 to April 2013 after the exposure of the research objectives to the patients. Participants were instructed on the objectives of the study and signed the Informed Consent Form (ICF). In case of minors under 18, the ICF was signed by the parent or guardian. Patients diagnosed with malaria were treated according to the Ministry of Health.

The DNA samples and the clinical data of the patients were provided by the TMF, and these samples were sent to the Genomics Laboratory at the Hematology and Hemotherapy Hospital Foundation of Amazonas-HEMOAM, where they were analysed through molecular techniques for the detection of Duffy polymorphisms. This is a cross sectional study which the diagnosis of malaria was carried out at the TMF in the state of Amazonas, Brazil, through a specialized team with a standardized clinical and laboratory method.

The diagnosis of malaria followed the standardized course of the TMH which is a national reference in the diagnosis and treatment of malaria. In this course a multiple reading of all the slides is carried out to confirm the parasitaemias, as well as the confirmation of the Plasmodium species detected by the polymerase chain reaction (PCR) method.

The 7.2% *FYB/FYES* percentage found in the study by Albuquerque et al. was used as the basis for the sample calculation. This was the lowest frequency found in a total of 15.4% of patients with alleles of greater impact on parasitic density (*FY/FYES, FY/FYW*). We also used to compose the calculation, the annual average of 3100 patients treated at the Institute of Tropical Medicine of Manaus Doctor Heitor Vieira Dourado, positive for *P. vivax*.

The sample was calculated using the program R Core Team statistical package (2013), using as parameters p probabilistic error of 0.05 and effect size of 0.5, with statistical power of 95%, so that the minimum sample determined was 122 individuals in total. Thus, the sampling of 287 was above the stipulated and thus we obtained an effect size and the statistical test increased [17].

**Fig. 1:**
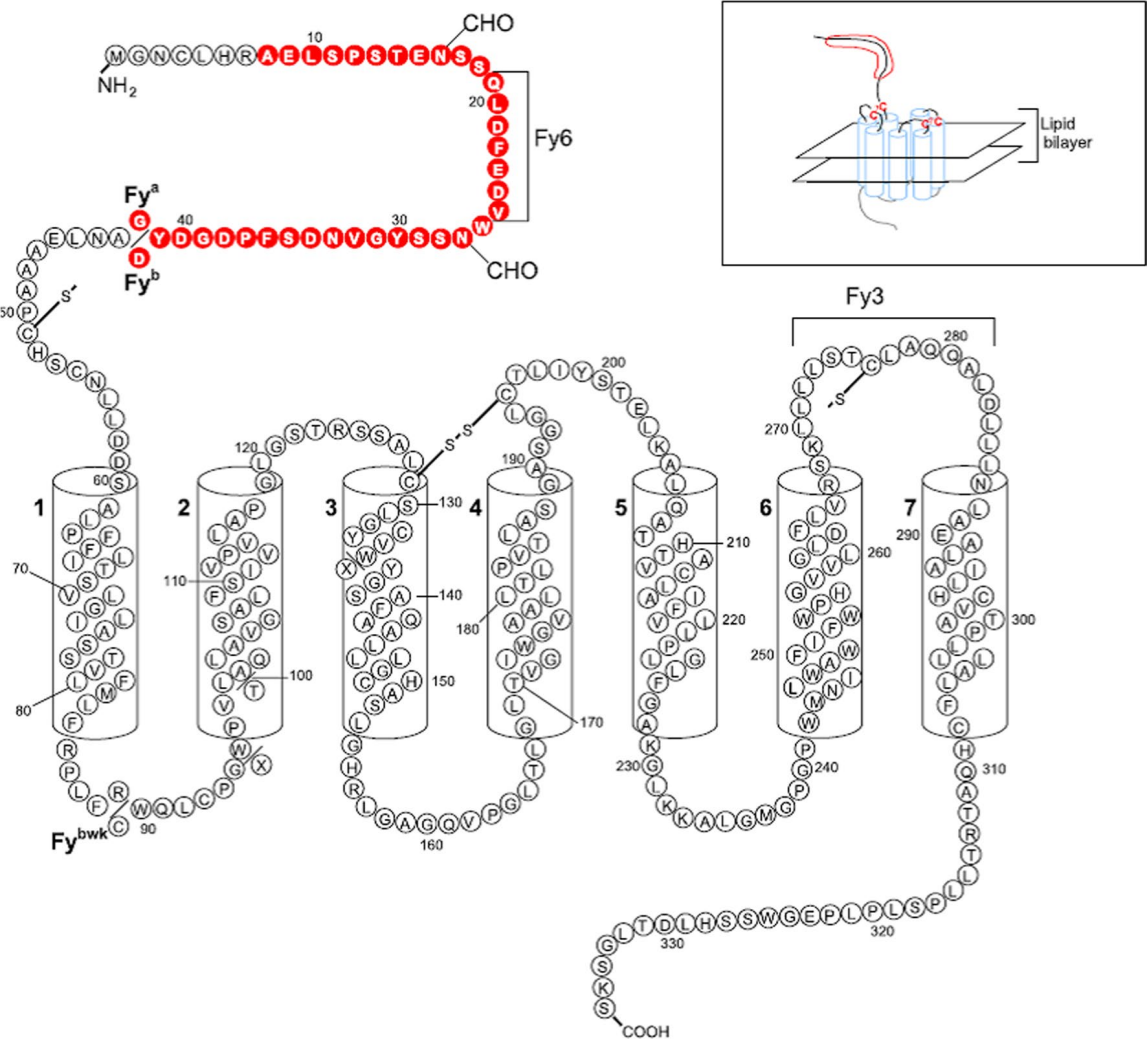
Demonstration of Duffy protein, emphasizing its antigens and amino acids [15]

Parasitaemia was determined using a standard parasitological method, the thick blood smear, which involves collecting a drop of blood from the finger on a glass slide, followed by staining with vital dyes such as methylene blue or Giemsa. This technique is based on visualization of the parasite by means of optical microscopy. After staining, specific differentiation of the parasites is possible by optical microscopy, based on the analysis of their morphology and stages of development in the peripheral blood (Fig. 1).

The determination of parasitaemia was based on the count of asexual parasites per 200 leukocytes and, in the case of less than 10 parasites identified in the reading, the count will continue up to 500 leukocytes. Microscopic counts greater than 500 parasites were interrupted after reading the last field (in which the number 500 occurred), even before reaching 200 leukocytes (Table 3).

**Table 3:** Semi quantitative evaluation of Plasmodium parasitic density by thick blood smear microscopy

Number of parasites counted/field	Qualitative parasitaemia	Quantitative parasitaemia (per mm^3^)
40 to 60 per 100 fields	+/2	200–300
1 per field	+	301–500
2–20 per field	++	501–10‚000
21–200 per field	+++	10‚001–100‚000
200 or more per field	++++	> 100‚000

Ministry of Health. Practical Guide to Malaria Treatment in Brazil. 1 Ed. 2010

The total number of white blood cells (leukocytes) of each patient was determined for the determination of the parasite density (PD). PD was calculated using the following formula:

$${\text{Parasite density}}/\mu {\text{L }} = \frac{{{\text{Number of parasites }} \times {\text{ Total white blood cells }}\left( {\text{WBC}} \right)\,{\text{counts}}}}{{{\text{Number of white blood cells }}\left( {\text{WBC}} \right)\,{\text{ counted}}}}$$

The microscopic diagnosis with negative result was defined when, in at least 300 fields of the slide, no asexual forms of *P. vivax* were found. The implementation of the thick smear, as well as the definitions of parasitaemias and parasite densities (PD), was carried out according to the protocols of the Amazon Network for Surveillance of Resistance to Antimalarial Drugs - RAVREDA [18]. For division of PD into low, medium and high, the classification described by Alecrim et al. was used [19].

This classification is based on counting parasites for every 100 leukocytes as described below:

- Low parasite density: counting up to 7000 trophozoites per µL;
- Average parasite density: trophozoites counts between 7001 and 15,000 per µL;
- High parasite density: trophozoites number equal or greater than 15.001 per µL.

The limits of these categories were defined in the study by Alecrim et al. comparing the amount of parasites found and the symptoms presented by the patients studied. The clinical symptoms of malaria may be mild, moderate or severe, depending on the species of the parasite, the amount of circulating parasites, the time of disease and the level of immunity acquired by the patient [4].

Duffy genotyping was performed at the HEMOAM of the State of Amazons, Brazil, using the Bead Chip BioArray system. For genotyping, DNA from the patients were extracted using the kit QIAmp^®^ Blood Mini kit (Qiagen, Hilden, Germany). The concentration and quality of DNA was determined by measuring optical density in a Thermo Scientific Nano Drop 2000 apparatus. Genotyping was then performed using BioArray Human Erythrocyte Antigens (HEA) BeadChip (Immucor, Peachtree Corners, Geórgia, EUA), a high-throughput molecular assay that detects 38 red blood cell antigens and phenotypic variants. The BeadChip contains all probes of interest including internal positive controls, and negative controls of the reaction. Each probe is covalently attached to a microsphere type that can be distinguished on the basis of their spectrum qualities. The fluorescent signals emitted by different microspheres are then captured by imaging system (AIS400). Results obtained were analysed using (BioArray Solutions Information System) BASIS™ (data analysis software).

In the determination of the Duffy system the following polymorphisms were analysed: *FYA/FYB* - SNP 125 G>A; GATA - (Fy silencing) - SNP - 67 T>C and *FY* weak - SNP 265 C>T.

## Results

**Fig. 2:**
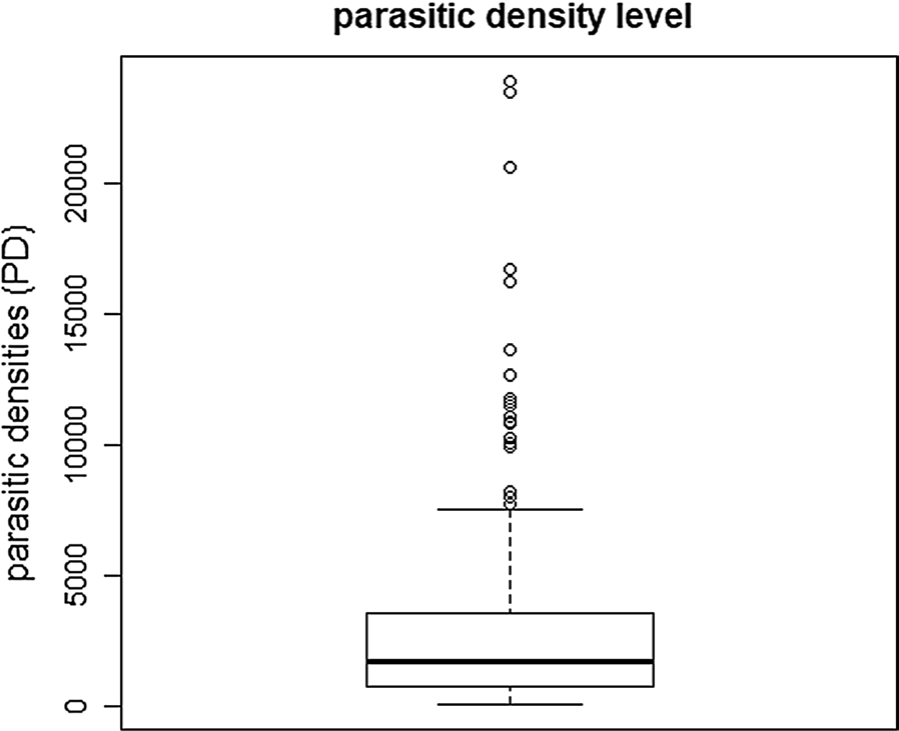
Level of parasite densities found in the studied patients

The levels of parasite density found in the studied patients considering the number of parasites found per mm^3^ is showed in the Fig. 2. Regarding age and gender of patients infected with *P. vivax*, it is possible to observe a statistically non-significant distribution in the Table 4.

**Table 4:** Distribution of gender and age of the participants

Age	Gender				Total	p valor
	Male		Female			
	n	%	n	%		
0–10	11	5‚5	08	9‚2	19	
11–20	22	11	13	15	35	
21–30	31	15‚5	09	10‚3	40	
31–40	52	26	14	16	66	> 0‚05
41–50	38	19	16	18‚4	54	
51–60	30	15	18	20‚7	48	
61–70	13	6‚5	06	07	19	
71–80	03	1‚5	03	3‚4	06	
Total	200	100	87	100	287	

Duffy genotyping was successfully performed on 287 patients as showed in Table 5 where it is possible to observe the distribution of the Duffy genotypes in the different levels of parasite density. In the studied samples, we did not find FYA*ES alleles, as well as FYA*W and even negative Duffy patients infected with *P. vivax*.

**Table 5:** Duffy genotypes of patients infected with vivax malaria according to their parasite density

Genotypes	Parasite density—DP								p valor
	Low		Medium		High		Total		
	n	%	n	%	n	%	n	%	
FY*01/FY*01	63	23‚7	04	25‚0	01	20‚0	68	23‚7	
FY*01/FY*02	114	42‚8	09	56‚3	01	20‚0	124	43‚2	
FY*01/FY*02N.01	23	8‚6	–	–	–	–	23	8‚01	< 0‚05
FY*01/FY*02W.01	01	0‚4	–	–	–	–	01	0‚34	< 0‚05
FY*02/FY*02	55	20‚7	03	18‚7	03	60‚0	61	21‚2	
FY*02/FY*02N.01	08	3‚0	–	–	–	–	08	2‚8	< 0‚05
FY*02/FY*02W.01	01	0‚4	–	–	–	–	01	0‚34	
FY*02N.01/FY*02W.01	01	0‚4	–	–	–	–	01	0‚34	
Total	266	100	16	100	05	100	287		

FY*01 (FYA); FY*02(FYB); FY*02N.01(FYB ^*ES*^ ); FY*02W.01(FYB ^*W*^ *)*

In all samples studied, both polymorphisms at position 256C>T of the DARC gene and the SNP-67 T>C in the GATA promoter region were found only associated with the FY*2 allele characterizing all polymorphic samples as FY*02W. 01 and FY*02.01 [20].

**Fig. 3:**
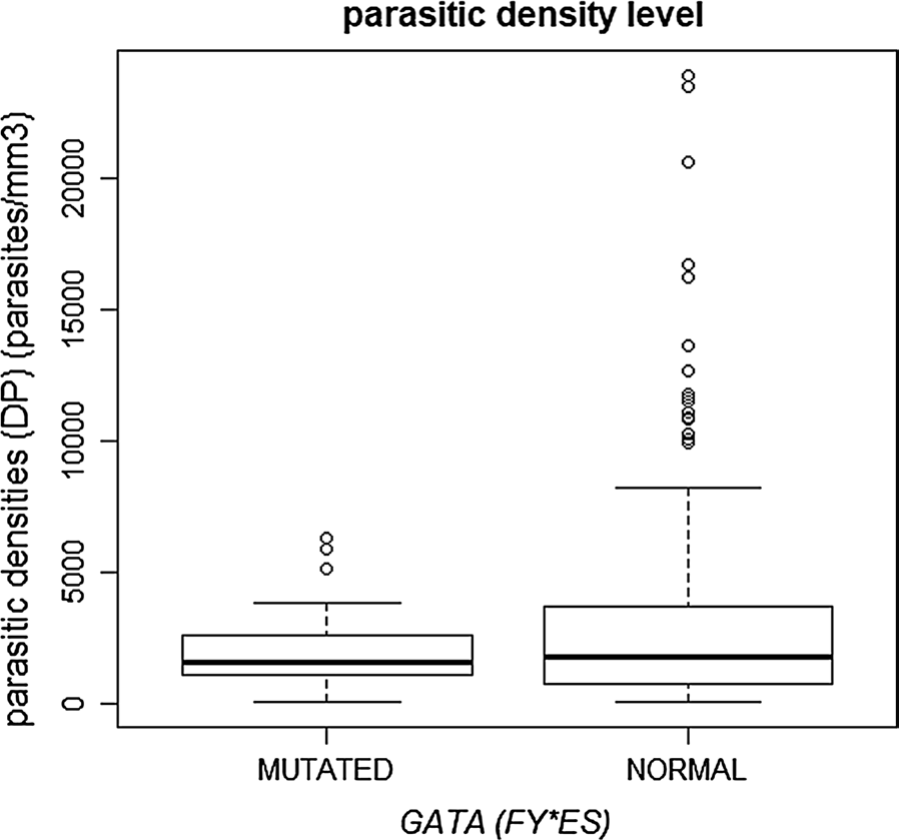
Level of parasite density found in patients with mutated GATA

**Fig. 4:**
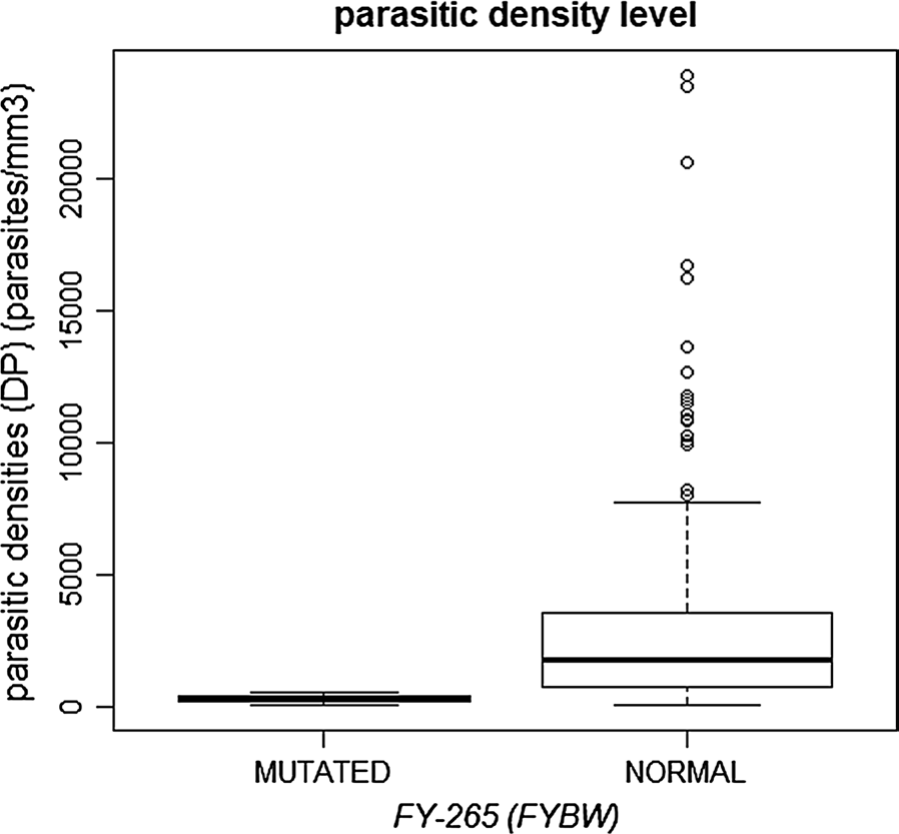
Level of parasite density found in patients with *FYBW*

The genotypes FY*01/FY*01; FY*01/FY*02 and FY*02/FY*02 were found in patients with three levels of parasite density (Low, Medium and High), but genotypes formed with FY*01 or FY*02 alleles in heterozygosity with FY*02N.01 and FY*02W.01 alleles, were found in 34 (11.8%) patients who presented only low parasite density as shown in Table 5 and Figs. 3 and 4. Another interesting finding found in this study was the presence of the genotype FY*02N.01/FY*02W.01 in one of those patients infected with *P. vivax* with the lowest parasite density found (78 parasites/mm^3^) presenting a type of malaria considered subclinical, being that this genotype, to date, had not yet been described in the literature.

Distribution of Duffy allele frequencies in respect of low, medium and high parasitic density is showed in the Table 6 and Figs. 2 and 3 with a statistic significance (p > 0.05%) related to the FY*02N.01 and FY*02W.01 alleles, low parasites density. It was made a search to found other associations relationing the Duffy mutated alleles, age and gender of the participants as showed in the Table 7, but it was not statistically significant.

**Table 6:** Frequency of alleles of patients infected with vivax malaria found according to parasite density

Alleles	Parasite density—DP						p valor
	Low		Medium		High		
	n	%	n	%	n	%	
FY*01	264	49‚6	17	53‚1	3	30‚0	
FY*02	233	43‚8	15	46‚8	7	70‚0	
FY*02N.01	32	6‚0	0	0	0	0	< 0‚05
FY*02W.01	3	0‚5	0	0	0	0	< 0‚05
Total	532	100	32	100	10	100	

FY*01 (FYA); FY*02(FYB); FY*02N.01(FYB ^*ES*^ ); FY*02W.01(FYB ^*W*^ *)*

**Table 7:** Distribution of mutated Duffy among the participants according they age and gender

Age	Gender							
	Male				Female			
	n	Duffy mutated	%	p	n	Duffy mutated	%	p
0–10	11	04	13‚8	> 0‚05	08	01	16‚6	> 0‚05
11–20	22	02	6‚9		13	00	–	
21–30	31	05	17‚2		09	00	–	
31–40	52	05	17‚2		14	00	–	
41–50	38	04	13‚8		16	04	66‚6	
51–60	30	06	20‚7		18	01	16‚6	
61–80	16	03	10‚3		09	00	–	
Total	200	29	100		87	06	100	

Obs: One of the 27-year-old male participants was heterozygote with one GATA and one FY 256 allele and parasitic density with 78 parasites per mm^2^

No female participant showed the mutation FY 265

All participants showing Duffy mutations had also low parasity density

One of the 27-year-old male participants was heterozygote with GATA and FY 256 allele and parasitic density with 78 parasites per mm^2^.

No female participant showed the mutation FY 265.

All participants showing Duffy mutations had also low parasity density.

### Duffy glycoprotein quantification

Flow cytometry to quantify Duffy glycoprotein expression in patients with low parasitic density could not be performed due to limitations of the institution’s technical and infrastructure for this purpose. However, reference was made to other studies (see Table 2) that show the reduction of expression in several genotypes found in this study. Although Duffy density was not quantified in the studied population, the proposed association between genotype, Duffy glycoprotein expression and reticulocyte invasion by *P. vivax* was performed only in a comparative and inferential manner.

## Discussion

One of the greatest difficulties encountered in the fight against malaria in the Amazon has been reported as subclinical malaria that occurs when the individual is infected but has no or few symptoms, being this a point that deserves additional studies. As these infected individuals were without symptoms, did not seek the medical service and, therefore, were not treated, they become undetectable transmitters of this disease [21].

The results showed no statistically significant association between demographic characteristics of participants, such as age or gender with *P. vivax* infection as well as the frequency of mutated Duffy alleles. The authors do not speculate on the race of the participants due to the strong regional ancestral mix found in the Amazonian caboclos, which originate with the arrival of Caucasians and blacks in indigenous lands.

All participants with the genotype composed of one of the polymorphic alleles as the mutated GATA (FY*02N.01) and weak duffy (FY*02W.01) alleles in heterozygosis with FY*01 or FY*02, showed low parasite density, demonstrating a possible reduction in *P. vivax* adhesion caused by the reduction of the Duffy glycoprotein due to the presence of the polymorphic alleles, as demonstrated by King et al. who support the hypothesis that erythrocytes, expressing the FY */FY*02N.01, have a significant reduction in parasite adhesion when compared to erythrocytes expressing FY*02/FY*02, [22].

Woolley et al. demonstrated in their study differences in the expression level of Fy^6^ epitopes on the surface of reticulocytes from individuals with Fy phenotype (a + b +) by the flow cytometry technique. This expression was significantly lower in reticulocytes and mature red blood cells (RBC) in the FY*02/FY*02 genotypes than in the FY*01/FY*01 or FY*01/FY*02 genotypes. The authors concluded that heterozygous individuals may have a greater amount of erythrocyte receptor variation for binding parasite proteins of *P. vivax* [23].

The results also agree with another very important study on the expression of the Duffy glycoprotein published by Tournamille et al. and Wolley et al. which showed that the presence of the FY*02N.01 allele results in a 50% reduction of that protein on the surface erythrocyte invasion by *P. vivax*, although Twohig et al. reported evidence of Duffy-negative individuals (FY*02N.01/FY*02N.01) in regions endemic in Africa. which may be linked to the very low expression of glycoproteins, parasite *P. vivax*-like or even the existence of an alternative gateway found by the parasite.

Yazdanbakhsh et al. demonstrated by flow cytometry a 10% reduction of the Duffy protein in the erythrocyte membrane of individuals with the heterozygous allele FY*02W.01, due to a protein instability caused by the Arg89Cys polymorphism, which probably explains the reduction of the parasitic density in cases found in the Brazilian Amazon. Although the initial finding was related to a reduction in the incidence of malaria and not to the density of the parasite, this aspect is also important to address since it may be related to sub-clinical or asymptomatic malaria as found in results of this study.

It was possible to find FY*02W.01 only in 3 patients who had a very low parasite density (78–513 trophozoites/μL), showing that this allele may be associated with subclinical malaria, confirming data published by Albuquerque et al. [24]. The parasite density is defined by the number of parasites found per microlitre of the patient’s blood and has been associated with the severity of malaria. A high or low parasite density may depend on factors involving parasitic invasion of *P. vivax* into erythrocytes, such as the Duffy glycoprotein polymorphisms in the host.

Cases of high parasite density related to *P. vivax* infection, sporadically reported in some parts of the world were not quantified. According to the results, these cases occurred particularly more frequently in patients with FY*02 allele, demonstrating that these variables may potentially be associated.

Another interesting finding was the presence of the FY*02N.01/FY*02W.01 genotype in one of the infected patients. This genotype was observed for the first time in the population studied. As expected, this patient had a very low parasite density, phenotyped as Duffy negative and presenting few symptoms of the disease. This genotype may be contributing to subclinical malaria in the Brazilian Amazon. However, only one individual was found, requiring a broader search for the confirmation of this new association study with malaria.

Kasehagen et al. conducted a cross-sectional malaria prevalence surveys in Papua New Guinea (PNG), where they have previously identified a new Duffy-negative allele among individuals living in a region endemic. This study provides the first evidence that Duffy-negative heterozygosis reduces erythrocyte susceptibility to *P. vivax* infection [25].

34 patients (11.8%) were found with a mutated Duffy allele that may have interfered with malaria infection, and in particular one patient with the FY*02N.01/FY*02W.01 genotype presenting the lowest density (78 parasites/mm^3^) and did not present symptoms of fever, chills and pain, among other characteristics of malaria, corresponding to 0.34% of the 287 patients studied. Despite the almost imperceptible symptomatology presented, this patient sought the medical service because relatives living in the same residence were infected and with symptoms characteristic of the disease. In a simple calculation of population projection based on these results, considering a region of approximately 12 million inhabitants living in an endemic area of malaria in the Brazilian Amazon, considering also the real statistical possibility of finding individuals with polymorphic duffy genotypes as described, it was infered that in addition to the Duffy negative individuals existing in the region, there is a considerable amount of inhabitants that may be serving as human reservoirs for the spread of malaria in the Brazilian Amazon making malaria eradication difficult.

## Conclusion

The present study presented new perspectives on the relationship of Duffy polymorphisms with parasitaemia and symptoms of malaria caused by *P. vivax*. The FY*02N.01 and FY*02W.01 alleles were found to be associated with low parasitic density in infected patients. Therefore, these polymorphisms appear to have a significant impact on the population in areas where malaria is endemic, and may lead to partial defense mechanisms against *P. vivax.*

## Data Availability

The electronic files, consent terms, database, as well as the materials used for interpretation, replication and findings can be found and made available at the Hematology and Hemotherapy Foundation of the State of Amazonas (HEMOAM) under the responsibility of the corresponding author and may be available upon reasonable request.

## Abbreviations

*FY*: representation of the allelic form of the Duffy system
*FY*01*: Duffy allele A
*FY*02*: Duffy allele B
*FY*02N.01*: Duffy B allele not expressed in the erythrocyte membrane because it has its mutated promoter region
*FY*02W.01*: Duffy B allele is weakly expressed in the erythrocyte membrane because it has polymorphisms in the nucleotide chain
*FYB*^*ES*^: Duffy B erythroid Silent
*FYB*^*W*^: Duffy B Weak
DARC: Duffy Antigen Receptor for Chemokines. Name of the gene responsible for the expression of the duffy blood group system
SNP: Single Nucleotide Polymorphism
GATA: promoter region that regulates DARC gene expression in erythrocytes
ICF: Informed Consent Form
µL: microlitre
WBC: white blood cell
RBC: red blood cell
PNG: Papua New Guinea
PD: parasite densities
HEA: Human Erythrocyte Antigen
BASIS: Bio Array Solutions Information System
ISBT: International Blood Transfusion Society
WHO: World Health Organization
PAHO: Pan American Health Organization
TMF: Tropical Medicine Foundation
HEMOAM: Hemocentro do Amazonas. Hematology and Hemotherapy Hospital Foudation of Amazonas State
RAVREDA: Rede Amazônica de Vigilância da Resistência a Drogas Anti-Malária. Amazonian Network for Surveillance of Anti-Malarial Drug Resistance
